# Influenza Vaccination among Multiple Sclerosis Patients during the COVID-19 Pandemic

**DOI:** 10.3390/vaccines10101766

**Published:** 2022-10-21

**Authors:** Ignacio Hernández-García, Moisés Garcés-Redondo, Judit Espinosa-Rueda, Joana Rodríguez-Montolio, Irantzu Bengoa-Urrengoechea, Carlos Aibar-Remón

**Affiliations:** 1 Department of Preventive Medicine and Public Health, Lozano Blesa University Clinical Hospital of Zaragoza, Calle San Juan Bosco 15, 50009 Zaragoza, Spain; 2 Health Services Research Group of Aragon (GRISSA), Aragon Institute for Health Research (IISA), Calle San Juan Bosco 15, 50009 Zaragoza, Spain; 3 Department of Neurology, Lozano Blesa University Clinical Hospital of Zaragoza, Calle San Juan Bosco 15, 50009 Zaragoza, Spain; 4 Department of Preventive Medicine and Public Health, University of Zaragoza, Calle de Pedro Cerbuna 12, 50009 Zaragoza, Spain

**Keywords:** multiple sclerosis, influenza vaccines, vaccination coverage, associated factors, Spain, COVID-19

## Abstract

In the context of the COVID-19 pandemic, the co-circulation of influenza and SARS-CoV-2 viruses may have severe complications for vulnerable populations. For this reason, the World Health Organization pointed to the 2020–2021 anti-influenza campaign as being of special relevance. Our aim was to assess the 2020–2021 influenza vaccination coverage, and its associated factors, among patients in a Spanish multiple sclerosis (MS) unit. A cross–sectional study was conducted. People attending the MS unit of the Clinical Hospital of Zaragoza during 2020 were included. Variables were obtained by reviewing records. Associations with 2020–2021 influenza vaccination were analyzed using bivariate analysis and a multiple logistic regression model. A total of 302 patients were studied; 62.6% were women, whose mean age (standard deviation) was 47.3 (11.5) years. The 2020–2021 influenza vaccination coverage was 55.3% (59.8% in women and 47.8% in men). A total of 89.7% had at least one other indication for vaccination (e.g., immunosuppressive treatment in 225 patients). The variables associated with getting vaccinated were being female (adjusted odds ratio (95% confidence interval) (aOR (95%CI) = 2.12 (1.12–3.99)), having received the 2019–2020 influenza vaccine (aOR (95%CI) = 31.82 (14.71–68.86)) and being born in Spain (aOR (95%CI) = 12.91 (1.07–156.28)). Coverage is moderate compared to other countries. It is necessary to develop strategies to improve it, especially in men and those born outside Spain.

## 1. Introduction

Globally, it is estimated that up to 650,000 people die each year from influenza-related causes [1]. In Spain, during the 2019–2020 season, 619,000 people attended primary healthcare consultations for influenza. There were 27,700 hospitalizations with laboratory test-confirmed influenza, 1800 admissions to intensive care units and 3,900 influenza-associated deaths [2]. The percentage of positive results remained above 40% for 11 consecutive weeks (week 51/2019 through week 9/2020) [3].

In particular, influenza can also cause serious complications in people with multiple sclerosis (MS), such as severe exacerbations of MS symptoms [4,5]. For this reason, public health institutions [6,7], scientific societies [8,9,10] and expert groups [5,11] recommend annual influenza vaccination for patients with MS. However, the vaccination coverage in this population is low [12,13,14,15,16,17], with the rates in Europe ranging from 19% in Germany [12] to 63.8% in Ireland [15] (Table 1), while in the Americas, they range from 45.4% in Latin America [16] to 59.1% in the USA [13].

In Spain, since the 2014–2015 season, the Ministry of Health has recommended annual flu vaccination for people with MS [18]. For this purpose, inactivated vaccines of proven effectiveness and safety are used. These vaccines are usually administered free of charge at primary healthcare centers [2].

In the context of the novel Severe Acute Respiratory Syndrome Coronavirus (SARS–CoV–2) pandemic [19], the cocirculation of influenza and SARS–CoV–2 viruses may have severe complications for vulnerable populations and can overload the health systems (in terms of the number of admissions and consultations) [7]. For this reason, the World Health Organization and the Spanish Ministry of Health pointed to the 2020–2021 anti-influenza campaign as being of special relevance [2,7].

This research was carried out with the objective of knowing the coverage of influenza vaccination in 2020–2021 and its associated factors in patients in a Spanish MS unit.

## 2. Materials and Methods

A cross-sectional study was conducted in Aragon (Spain); about 1,200,000 people live in this Spanish region. Among them, about 1300 have MS [20]. The University Clinical Hospital of Zaragoza (UCHZ) houses one of the two MS units in Aragon; in it, patients with MS are monitored physically and/or remotely at least once every six months. Approximately 25% of MS patients living in Aragon are seen in such unit.

Patients seen in the MS unit of the UCHZ between 1 January 2020 and 31 December 2020 were included. The exclusion criterion was death before the start of the 2020–2021 influenza campaign (15 October 2020).

In October 2021, physicians of the MS unit of the UCHZ obtained the following information by reviewing electronic medical records from both specialized care and primary care: sex, country of birth, date of birth, place of residence (city of Zaragoza or other), allergies, date and age at MS diagnosis, MS type (relapsing–remitting, secondary progressive, clinically isolated syndrome or primary progressive) [21], number of MS outbreaks during 2019, Expanded Disability Severity Scale (EDSS) score [22] during 2020, having received the 2019–2020 flu vaccine, registration of the recommendation to get an annual flu vaccination at a primary healthcare center, date and place of administration of the 2020–2021 influenza vaccine, and belonging to any other influenza vaccination target group according to the recommendations of the Spanish Ministry of Health (e.g., (a) 65 years of age or older, (b) chronic cardiovascular disease, (c) isolated arterial hypertension, (d) chronic respiratory disease, (e) diabetes mellitus, (f) morbid obesity, (g) chronic kidney disease, (h) hemoglobinopathies and anemias, (i) asplenia, (j) chronic liver disease, (k) immunosuppressive treatment, (l) cancer, (m) cochlear implantation or awaiting cochlear implantation, (n) disorders and diseases leading to cognitive dysfunction, (o) cerebrospinal fluid fistula, (p) celiac disease, (q) intestinal inflammatory disease, or (r) pregnant in any gestational trimester) (Table S1) [18]. Moreover, information on pneumococcal vaccination status (pneumococcal conjugated vaccine 13–valent (PCV13) and pneumococcal polysaccharide vaccine 23–valent (PPSV23)) until 31 December 2020 was collected in patients taking immunosuppressive medication.

For the statistical analysis, we used measurements of central tendency (mean or median) and dispersion (standard deviation (SD) or range) for quantitative variables (according to whether or not they presented a normal distribution according to the Kolmogorov–Smirnov normality test) and absolute and relative frequencies (percentages) for qualitative variables. The bivariate analyses considered having received the 2020–2021 flu vaccine as the dependent variable and the others as independent variables using the chi square test or the Fisher exact test.

Subsequently, a multiple logistic regression analysis was performed with the variables for which significant associations were observed in the bivariate analyses. To quantify the associations, the adjusted odds ratio (aOR) was calculated with its 95% confidence intervals (95%CI). 

Moreover, in patients taking immunosuppressive medication, bivariate analyses considered having received the 2020–2021 flu vaccine as the dependent variable and having received the pneumococcal vaccination (PCV13 plus PPSV23) as the independent variable in the chi square test.

A value of 0.05 was taken as the alpha probability of error. All of this was carried out using the analysis program SPSS v.24.0 (IBM Corp, Hong Kong, China). This study was approved by the Research Ethics Committee of the Autonomous Community of Aragon (approval code C.P.–C.I. PI21/417).

## 3. Results

The number of persons included was 302, after excluding 1 patient due to death in July 2020. A total of 62.6% (189/302) were women, with a mean age (SD) of 47.3 (11.5) years during the 2020–2021 influenza vaccination campaign. Of the patients, 96.3% were born in Spain, and 53.6% resided in the city of Zaragoza. The median age (range) at diagnosis of MS was 31 (10–62) years. A total of 75.2% (227/302) had relapsing–remitting MS. During 2019, one or more MS outbreaks were suffered by 9.3% (28/302) of people. None of the patients were allergic to the components of the influenza or pneumococcal vaccines. A total of 89.7% (271/302) had at least one other indication for flu vaccination (the most frequent was immunosuppressive treatment, in 225 patients) (Table 2).

The flu vaccination coverage in 2020–2021 was 55.3% (167/302); by sex, the coverage was 59.8% in women and 47.8% in men. Depending on the country of birth, the coverage ranged from 57.1% in those born in Spain to 9.1% in those born outside Spain. All flu vaccines were administered at primary healthcare centers.

In the bivariate analyses, the variables associated with being vaccinated against 2020–2021 flu were: female sex, history of influenza vaccination in 2019–2020, type of MS, EDSS score, having received the recommendation for annual influenza vaccination at primary healthcare centers, having diabetes mellitus and being born in Spain (Table 3). 

In the logistic regression analysis, the variables that maintained their significant association were being female (aOR (95%CI) = 2.12 (1.12–3.99)), having received the 2019–2020 flu vaccine (aOR (95%CI) = 31.82 (14.71–68.86)) and having been born in Spain (aOR (95%CI) = 12.91 (1.07–156.28)) (Table 4).

In patients taking immunosuppressive medication, an association was detected between being vaccinated against influenza in 2020–2021 and a history of pneumococcal vaccination (PCV13 plus PPSV23) (*p* < 0.001). Thus, 53.2% (66/124) of those vaccinated for influenza in 2020–2021 had a history of vaccination against pneumococcus, while 26.7% (27/101) of those not vaccinated against influenza in 2020–2021 had a history of vaccination against pneumococcus.

## 4. Discussion

This is, to our knowledge, the first study to evaluate the 2020–2021 influenza vaccination coverage in people with MS in Spain. The rate obtained is moderate with respect to those documented internationally [12,13,15,16,17,23,24], where, in the 2020–2021 season, coverages of 45.4% [16] to 63.8% [15] and 68.6% [24] were described in persons with MS in Latin America [16], Ireland [15] and Italy [24], respectively.

Likewise, the rate obtained (55.3%) is higher than that described to date in Spain in patients with MS (from 20.4% in the 2016–2017 season to 41.5% in the 2019–2020 season) [14]. However, such rate is lower than that achieved in Spain during the 2020–2021 campaign in other groups targeted for vaccination, such as healthcare personnel (62.0%), those over 65 years of age (67.7%) and pregnant women (61.9%) [25]. Similarly, the rate obtained is lower than that achieved in other countries during the 2020–2021 campaign in healthcare personnel (75.9% in the USA [26] and 58.9% in Italy [27]) or those over 65 years of age (75.2% in the USA [28], 59.9% in France [29] and 73.5% in Greece [29]), although it was somewhat higher than that documented in pregnant women in the USA (54.5%) [30].

The corresponding percentage of unvaccinated patients (44.7%) is relevant given that, in addition, none had an allergy to the vaccine and almost 90% had at least one other indication for influenza vaccination. This could reflect the lack of awareness among people with MS about the importance of receiving this vaccine. In fact, in studies that have evaluated the hesitancy over the flu vaccine among people with MS, the most common reason they stated for not getting vaccinated was a perceived lack of necessity (the reason given by up to 50.0% of those not vaccinated) [15].

In contrast to other studies conducted prior to the coronavirus pandemic (COVID-19) caused by SARS-CoV-2 [14,15], having received the recommendation by healthcare personnel to be vaccinated was not associated with receiving the flu vaccine in 2020–2021. This may indicate that the context of the COVID-19 pandemic has contributed to patients who have not received this advice and who have not been vaccinated to date deciding to get vaccinated in the 2020–2021 season. Whether this change will continue in other seasons, along with the reasons for vaccination and non-vaccination in the new context of the COVID-19 pandemic, should be evaluated in further research in order to plan improvement strategies for this type of patients.

Meanwhile, it is necessary to implement some interventions aimed at improvement, such as facilitating the accessibility of the vaccine by offering it to patients when they come to the hospital. Thus, several authors have described how improving accessibility to the vaccine is a useful strategy to increase influenza vaccination coverage in other vaccination target groups [31].

The association between being vaccinated and having received the influenza vaccine in the previous season is consistent with that described in studies of other types of patients, in which previous vaccination has been described as an important predictor of flu vaccination [32]. The differences detected in influenza vaccination coverage according to sex (59.8% in women and 47.8% in men) represent an important reversal of the gender gap described so far in Spain, given that men tended to be vaccinated against influenza more frequently than women, regardless of their level of education or place of residence [33]. Thus, in the 2014–2015 season, differences in vaccination coverage by sex of up to 13.4 percentage points were documented in Spain among men aged 80 and over compared to women in that age group [33]. Similar differences have been documented in other types of patients [34,35,36], such as splenectomized patients (with flu vaccination coverage rates of 63.3% in men and 50% in women) [34] or individuals with chronic obstructive pulmonary disease (with vaccination rates of 62.6% in men and 53.6% in women in the 2012–2013 campaign) [36]. A contributing factor to this unexpected finding is perhaps the pandemic of the new SARS-CoV-2, in which influenza vaccination to avoid possible coinfection with the two viruses may have been a particularly important reason for women to be vaccinated.

As in Yap et al. [15], in the bivariate analysis, a higher EDSS score was associated with influenza vaccination. However, those authors only performed a bivariate analysis, without adjusting for other variables, as we have done in the multiple logistic regression analysis. Thus, the validity of comparing our results with those is limited.

On the other hand, the finding that those born outside Spain have been vaccinated significantly less than those born in Spain represents a health inequity (i.e., an unfair distribution of resources [37]). This inequity has also been described in other countries, such as Australia [38] and Italy [39], where flu vaccination coverage ranges from 16.9% among immigrants to 40.2% among Italian citizens [39]. Given the unfair nature of health inequities [37], it is especially necessary to develop specific strategies to increase vaccination among people born outside Spain. To this end, a specific investigation should be carried out to find out the reasons why these people are undervaccinated.

In Spain, since 2018, the Ministry of Health has recommended vaccination against pneumococcus (PCV13 plus PPSV23) in people taking immunosuppressive treatments [40]. In particular, in our study, among patients with MS who were taking immunosuppressive treatment, a history of pneumococcal vaccination (PCV13 plus PPSV23) was associated with influenza vaccination in 2020–2021. A similar association has been described by several authors in studies performed before the COVID-19 pandemic in other population groups. Domínguez et al. in Spain [41] and Hottes et al. in Canada [42] described how people aged 65 years or older who reported having received the influenza vaccination at least once were significantly more likely to report having received the PPSV23 vaccine. This may be because patients who follow the pneumococcal vaccination recommendation are more likely to accept other vaccines, such as the 2020–2021 influenza vaccine. However, we cannot confirm this, because the study of patient attitudes was not an objective of our research.

## 5. Limitations and Strengths

Among the limitations of this study is the sample size (302), which, although larger than the sample sizes in other studies [15,17,23,24] (from 101 [23] to 194 [17]), may have led to imprecise results. Nevertheless, the sample size is greater than 291, which is the minimum number to be studied considering a confidence level of 95%, a precision of 5%, a population of 1300 people [20] and an expected vaccination rate of 41.5% [14]. Although our research was conducted in an MS unit, it provides an example of a systematic approach to the evaluation of flu vaccination coverage in MS patients that could be carried out in other MS units [14]. Despite the fact that this was a registry study, there were no missing data about vaccinations because such information is always recorded in the electronic medical record (in its vaccines section). This is the first study to evaluate 2020–2021 influenza vaccination coverage in people with MS in Spain.

## 6. Conclusions

In Spain, in the COVID-19 era, influenza vaccination coverage in people with MS must be improved. To this end, it is particularly necessary to develop strategies, especially aimed at men and those born outside Spain.

## Figures and Tables

**Table 1:** Influenza vaccination rate among people with MS in European countries.

Country	Influenza Season	Vaccination Rate
Germany [12]	2017–18	19%
Spain [14]	2015–16	20.40%
	2016–17	20.40%
	2017–18	30.80%
	2018–19	41.20%
	2019–20	41.50%
Ireland [15]	2020–21	63.80%
Italy [17]	2020–21	58.20%

**Table 2:** Results of the descriptive analysis.

	*N* = 302
**Sex, *n* (%)**	
Female	189 (62.6)
Male	113 (37.4)
**Place of residence, *n* (%)**	
City of Zaragoza	162 (53.6)
Other	140 (46.4)
**Country of birth, *n* (%)**	
Spain	291 (96.3)
Morocco	2 (0.7)
Bulgaria	2 (0.7)
Other	7 (2.3)
**Multiple sclerosis type, *n* (%)**	
Relapsing–Remitting	227 (75.2)
Secondary Progressive	53 (17.5)
Clinically Isolated Syndrome	13 (4.3)
Primary Progressive	9 (3.0)
**Expanded Disability Severity Scale score, *n* (%)**	
0–1	123 (40.7)
1.5–3	85 (28.2)
3.5–6	46 (15.2)
6.5 or higher	46 (15.2)
Unknown	2 (0.7)
**Flu vaccination, 2019–2020, *n* (%)**	
No	177 (58.6)
Yes	125 (41.4)
**Recommendation for annual flu vaccination, *n* (%)**	
No	183 (59.6)
Yes	124 (40.4)
**Belonging to another group invited for influenza vaccination, *n* (%)**	
Yes	271 (89.7)
No	31 (10.3)

Results expressed as absolute (*n*) and relative (%) frequencies. “Recommendation for annual flu vaccination” means that, in the electronic medical record, it was written that you have been advised to have an annual flu vaccination.

**Table 3:** Results of bivariate analyses.

	Flu Vaccination in 2020–2021		*p*
	Yes (*n* = 167)	No (*n* = 135)	
**Sex, *n* (%)**			
Female	113 (67.7)	76 (56.3)	0.042
Male	54 (32.3)	59 (43.7)	
**Age during 2020–2021 campaign, *n* (%)**			
65 years of age or over	18 (10.8)	7 (5.2)	0.079
Under 65 years of age	149 (89.2)	128 (94.8)	
**Country of birth, *n* (%)**			
Spain	166 (99.4)	125 (92.6)	0.003
Other	1 (0.6)	10 (7.4)	
**Place of residence, *n* (%)**			
City of Zaragoza	84 (50.3)	78 (57.8)	0.195
Other	83 (49.7)	57 (42.2)	
**Multiple sclerosis type, *n* (%)**			
Relapsing–Remitting	120 (71.9)	107 (79.2)	0.047
Clinically Isolated Syndrome	4 (2.4)	9 (6.7)	0.025
Primary Progressive	7 (4.2)	2 (1.5)	0.709
Secondary Progressive	36 (21.5)	17 (12.6)	
**Multiple sclerosis outbreaks in 2019, *n* (%)**			
1 or more	17 (10.2)	11 (8.1)	0.545
None	150 (89.8)	124 (91.9)	
**Expanded Disability Severity Scale score, *n* (%)**			
6.5 or higher	31 (18.6)	15 (11.3)	0.039
3.5–6	32 (19.2)	14 (10.5)	0.02
1.5–3	43 (25.7)	42 (31.6)	0.888
0–1	61 (36.5)	62 (46.6)	
**Recommendation for annual flu vaccination, *n* (%)**			
Yes	85 (50.9)	39 (28.9)	<0.001
No	82 (49.1)	96 (71.1)	
**Flu vaccination in 2019–2020, *n* (%)**			
Yes	116 (69.5)	9 (6.7)	<0.001
No	51 (30.5)	126 (93.3)	
**Immunosuppressive treatment, *n* (%)**			
Yes	124 (74.3)	101 (74.8)	0.911
No	43 (25.7)	34 (25.2)	
**Cognitive dysfunction, *n* (%)**			
Yes	3 (1.8)	0 (0)	0.256
No	164 (98.2)	135 (100)	
**Chronic respiratory disease, *n* (%)**			
Yes	6 (3.6)	9 (6.7)	0.222
No	161 (96.4)	126 (93.3)	
**Chronic kidney disease, *n* (%)**			
Yes	4 (2.4)	1 (0.7)	0.385
No	163 (97.6)	134 (99.3)	
**Diabetes mellitus, *n* (%)**			
Yes	11 (6.6)	2 (1.5)	0.03
No	156 (93.4)	133 (98.5)	
**Chronic cardiovascular disease, *n* (%)**			
Yes	5 (3.0)	1 (0.7)	0.23
No	162 (97.0)	134 (99.3)	
**Isolated arterial hypertension, *n* (%)**			
Yes	25 (15.0)	12 (8.9)	0.109
No	142 (85.0)	123 (91.1)	
**Pregnancy, *n* (%)**			
Yes	3 (1.8)	0 (0)	0.256
No	164 (98.2)	135 (100)	
**Celiac disease, *n* (%)**			
Yes	2 (1.2)	3 (2.2)	0.659
No	165 (98.8)	132 (97.8)	
**Inflammatory bowel disease, *n* (%)**			
Yes	4 (2.4)	0 (0)	0.131
No	163 (97.6)	135 (100)	

**Table 4:** Variables included in the multiple logistic regression model.

	Flu Vaccination in 2020–2021		aOR (95%CI)	*p*
	Yes (*n* = 167)	No (*n* = 135)		
**Sex, *n* (%)**				
Female	113 (67.7)	76 (56.3)	2.12 (1.12–3.99)	0.02
Male	54 (32.3)	59 (43.7)	1	
**Country of birth, *n* (%)**				
Spain	166 (99.4)	125 (92.6)	12.91 (1.07–156.28)	0.046
Other	1 (0.6)	10 (7.4)	1	
**Flu vaccination in 2019–2020, *n* (%)**				
Yes	116 (69.5)	9 (6.7)	31.82 (14.71–68.86)	<0.001
No	51 (30.5)	126 (93.3)	1	

aOR (95%CI): adjusted odds ratio (95% confidence interval).

## Data Availability

The data are contained within the article.

## Supplementary Materials

The following supporting information can be downloaded at: https://www.mdpi.com/article/10.3390/vaccines10101766/s1, Table S1: 2020–2021 influenza vaccine recommendations. Spanish Ministry of Health.
