# Supplementary material for: Influenza Vaccination among Multiple Sclerosis Patients during the COVID-19 Pandemic
Source: Vaccines (Basel). 2022 Oct 21;10(10):1766. doi: 10.3390/vaccines10101766 (PMC9612003; doi:10.3390/vaccines10101766)
Supplement: Supplementary file 1 [file vaccines-10-01766-s001.zip › vaccines-1898128-supplementary.pdf]

Table S1. 2020-2021 influenza vaccine recommendations. Spanish Ministry of Health.

Persons 65 years or older
Persons less than 65 years who are at high risk of complications by influenza:
Minors (aged 6 months and older) and adults with chronic cardiovascular disease, isolated arterial hypertension, chronic neurological disease or chronic respiratory disease
Minors (aged 6 months and older) and adults with:
diabetes mellitus
morbid obesity
chronic kidney disease and nephrotic syndrome
hemoglobinopathies and anemias
hemophilia, other coagulation disorders, and chronic bleeding disorders, as well as recipients of blood products and multiple transfusions
asplenia or severe splenic dysfunction
chronic liver disease
severe neuromuscular diseases
immunosuppression
cancer
cochlear implantation or awaiting cochlear implantation
cerebrospinal fluid fistula
celiac disease
disorders and diseases leading to cognitive dysfunction (Down Syndrome, dementia or cognitive impairment)
intestinal inflammatory disease and inflammatory arthropathies -systemic erythematosus lupus, rheumatoid or juvenile arthritis-
Children between 6 months and 18 years of age, receiving prolonged treatment with acetylsalicylic acid
Persons of any age (≥6 months) institutionalized on a prolonged basis
Children between 6 months and 2 years of age with a history of prematurity less than 32 weeks of gestation
Pregnant women in any gestational trimester
People who can transmit influenza to those who are at high risk for complications:
Healthcare workers
Students in internships in healthcare centers
Persons providing home care to high-risk or elderly patients
Other groups in which vaccination is recommended:
People working in essential public services (State security forces and corps, firefighters, civil protection services and prison staff)
Persons with direct occupational exposure to domestic fowl or pigs and also to wild birds
